# How to solve the problem of inherited behavior patterns and increase the sustainability of technological civilization

**DOI:** 10.3389/fpsyg.2025.1562943

**Published:** 2025-02-13

**Authors:** Olev Vinn

**Affiliations:** Institute of Ecology and Earth Sciences, University of Tartu, Tartu, Estonia

**Keywords:** evolutionary behavior, inherited behavior patterns, sustainability of technological civilization, genetic engineering of behavior, Fermi paradox

## Introduction

All intelligent biological organisms in the universe could initially possess a range of genetically inherited behavior patterns (IBPs) that are not well-suited for the conditions of civilized societies (Vinn, 2024). These drives evolved to help species survive in their natural habitat, which offered a totally different context from that of a technological civilization. In fact, some of these IBPs may be highly incompatible with technological civilization and have the potential to lead to self-destruction (Vinn, 2024). Human civilization is shaped by various inherited behavior patterns (IBPs), many of which form the basis of human values, such as leadership (status within a group; Garfield et al., 2019; van Kleef and Cheng, 2020; Mitchell et al., 2020) and material wealth (control over energy resources; Chen, 2018; Mussel and Hewig, 2019). However, some of these IBPs are not well-suited to modern society and can result in negative consequences. For instance, the drive to acquire and display dominance over energy resources and social status can contribute to the overconsumption of resources, leading to ecological crises, and violent conflicts between groups, such as wars (Vinn, 2024). Other drivers of human behavior (Crusio, 2015; Plomin et al., 2016), while generally less harmful, still present risks. There is a strong genetic component among the controls on human behavior (Crusio, 2015). Plomin et al. (2016) found that all psychological traits show significant and substantial genetic influence and heritability is caused by many genes of small effect while no behavioral traits are 100% heritable. The less harmful drivers of human behavior include curiosity (Kidd and Hayden, 2015), which may prompt the premature use of dangerous technologies; the sex drive (Calabrò et al., 2019), which can lead to overpopulation; parental instincts (focused on nurturing offspring; Swain et al., 2014), and the desire for shelter (nesting; Chapin, 1951), which can push individuals to acquire disproportionately large shares of resources, leading to further conflict (Vinn, 2024).

The stability of complex ecological networks is influenced by both the interactions between species and the direct effects species have on themselves. These self-effects are referred to as “self-regulation,” which occurs when an increase in a species' population reduces its per capita growth rate (Barabas et al., 2017). Factors contributing to self-regulation include intraspecific interference, cannibalism, the separation of time scales between consumers and their resources, spatial heterogeneity, and non-linear functional responses that link predators to their prey (Barabas et al., 2017). The problems we face in technological civilization are so different from those in the natural habitats of human ancestors that they do not trigger evolutionary regulatory mechanisms in the right way, and most likely incompatible IBP-s do not work up to a specific limit.

**Figure 1:**
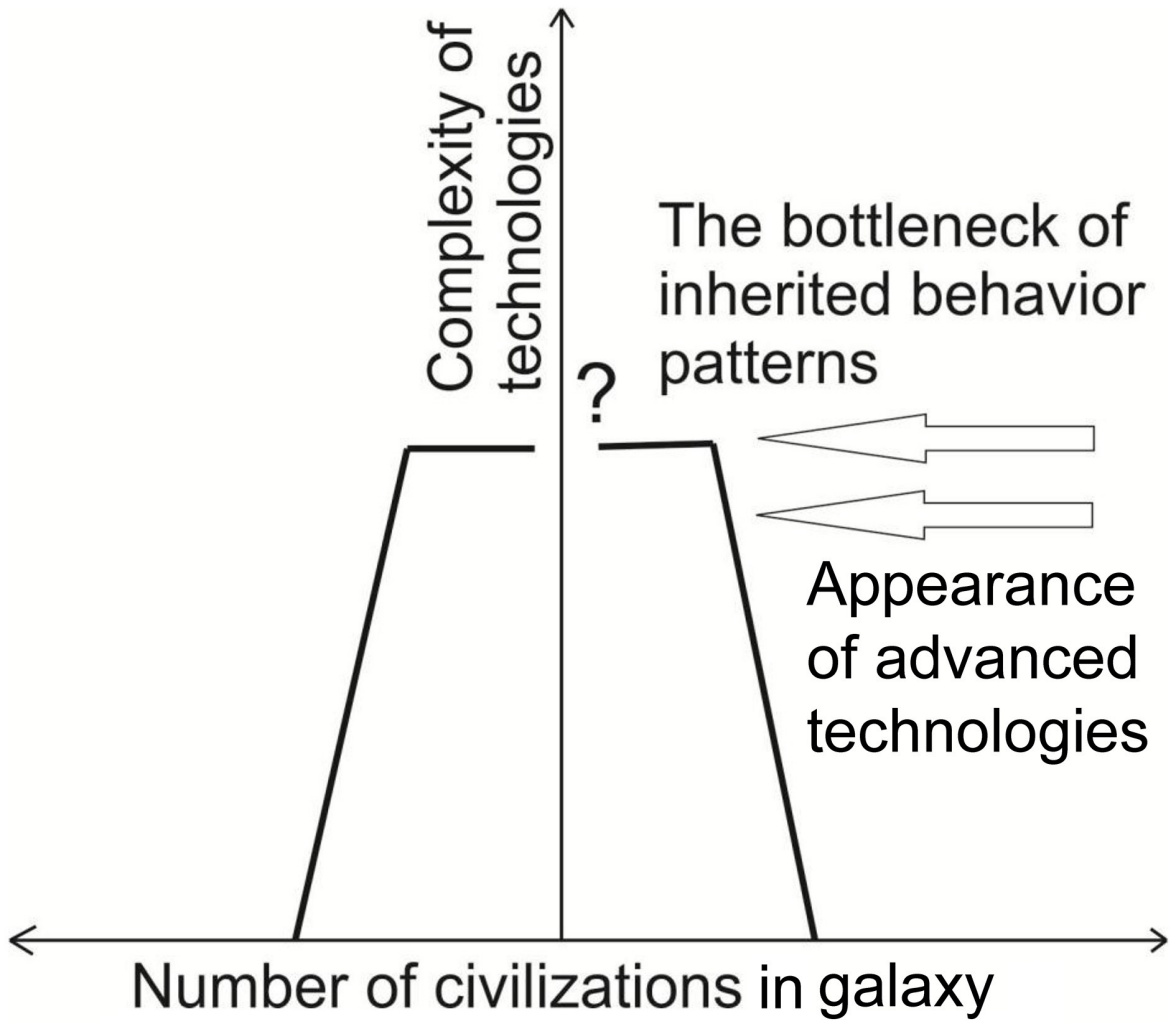
Possible dynamics of a number of civilizations in the galaxy. The extinctions before the bottleneck of IBPs are due to other causes (modified after Vinn, 2024, Figure 1).

According to Vinn (2024), incompatible inherited behavior patterns (IBPs) could present challenges that all emerging intelligences and technological civilizations must confront, which may help explain the Fermi paradox, at least in part, why we have not yet detected any alien civilizations (Figure 1). Vinn (2024) suggested that emerging civilizations that cannot solve the problem of incompatible IBPs may inevitably become extinct shortly after the appearance of advanced technologies.

## The current situation in dealing with incompatible IBPs

Human culture is the main mechanism that controls human social instincts (Boyd and Richerson, 2006). Human societies are exceptionally cooperative compared to most other animal species. The sick are tended to, and sharing results in significant food flows from the middle-aged to both the young and the elderly (Boyd and Richerson, 2006). Division of labor and trade are central aspects of every known human society (Boyd and Richerson, 2006). While violent conflict between large groups is frequent, all human societies are governed by shared moral systems that define individuals' rights and duties, typically enforced, though not always perfectly, by third-party sanctions (Boyd and Richerson, 2006). Among other major countermeasures that current technological civilization uses to mitigate the adverse effects caused by IBPs are religious in nature, particularly in the Western world. Christianity condemns the excessive need for energy resources, labeling it as the sin of greed (Mc Guinness, 1981). It also condemns the killing of conspecifics, which can be seen as the foundation for pacifism. Christianity does not endorse the pursuit of excessive power either. Among primate species, 29% form pair bonds, but since chimpanzees and bonobos do not, it's likely that the common ancestor of chimpanzees and humans also did not form pair bonds (Larsen, 2023). Henrich (2020) explained how the Church's medieval enforcement of lifelong monogamy, which deviated from the polygynous social structures of antiquity, played an important role in the prosperity of Western societies. In contrast, in Western capitalist societies, the need to possess excessive material resources is celebrated and termed entrepreneurship. Individuals who accumulate irrationally large amounts of wealth, such as billionaires, are often presented as positive role models for students in schools and universities (Jin et al., 2023). The desire for power (van Kleef and Cheng, 2020) is viewed as leadership and considered a positive trait. As a result, the current Western educational system is more likely to exacerbate the problem than to mitigate the outcomes of IBP-driven decision-making. The consequences include the over-exploitation of natural resources, ecological crises, and widespread violence, such as wars. The former is driven by the excessive desire for energy resources or, in religious terms, greed (Chen, 2018; Mussel and Hewig, 2019); the latter stems from IBP-driven leadership behaviors, or more specifically, the excessive need for power (van Kleef and Cheng, 2020). While the religious stance on some of the most dangerous IBPs is accurate, it seems ineffective in preventing the collapse of civilization. The same applies to cultural controls on dangerous IBP-s. On the other hand, the Western educational system has largely failed to suppress IBP-driven dangerous behaviors. In summary, current technological civilization is addressing the dangers posed by IBPs too ineffectively to ensure the long-term survival of civilization.

## Discussion

### What can be done more currently?

One of the first steps is to increase awareness of the threats posed by incompatible inherited behavior patterns (IBPs). This can be achieved through widespread education, aimed at both the general population and key decision-makers (Jin, 2014).

Education must emphasize the understanding of IBPs, their origins, and their potential consequences in modern societies (Griskevicius et al., 2012). It should not only focus on the harmful effects of IBPs but also promote strategies to mitigate them, especially in the context of modern technological and ecological challenges. This could include the incorporation of evolutionary psychology into educational curricula, helping individuals understand how our natural instincts often conflict with the needs of a sustainable civilization (Griskevicius et al., 2012).

Additionally, we can encourage the development and implementation of alternative value systems that better align human behavior with the species' long-term survival and the planet's health. This might involve promoting values like cooperation, sustainability, and empathy over the traditional emphasis on competition, accumulation of wealth, and power. Such shifts could be reinforced through social policies and cultural initiatives (Jin, 2014).

The role of leadership will be crucial in this process (Liao, 2022). Political and business leaders, educators, and influencers should actively model behaviors that reflect the awareness of the dangers posed by IBPs. They must prioritize long-term sustainability over short-term gains and encourage others to follow suit. In this way, leadership can become a tool for reshaping societal values and directing collective efforts toward solutions that align with the survival and flourishing of civilization (Liao, 2022).

Another key approach is the use of technology itself as a means to regulate and offset the negative impacts of IBPs. Technology can assist in human decision-making (Darioshi and Lahav, 2021). For example, we can develop systems of artificial intelligence and data-driven governance that monitor and guide societal behaviors, ensuring that decisions made at both individual and collective levels are more aligned with sustainability goals. Such systems could analyze trends and predict potential outcomes of current actions, allowing for more informed decision-making that takes into account the broader consequences for the environment, social stability, and future generations.

Finally, fostering a global sense of interconnectedness is essential (Hernández Guzmán and Hernández García de Velazco, 2023). As human beings become more aware of the shared nature of the planet's resources and the interdependence of all nations, it may become easier to shift away from self-destructive IBP-driven behaviors. Promoting global cooperation, especially in resource allocation and environmental conservation is what we can do now.

### Future solutions

Looking toward the future, one potential solution to the problem of incompatible inherited behavior patterns (IBPs) is genetic reprogramming (de la Torre and Chin, 2021), which could be developed over the next 50 to 100 years. Through advanced genetic engineering techniques (Tamura and Toda, 2020), we could potentially modify human behavior to be better suited for the challenges of a technological and sustainable civilization. Such genetic alterations would aim to reduce the negative impacts of IBPs, such as the excessive drive for power (Garfield et al., 2019; van Kleef and Cheng, 2020; Mitchell et al., 2020), greed (Chen, 2018; Mussel and Hewig, 2019), and overconsumption (Pratarelli, 2016), by reshaping the underlying instincts that drive these behaviors. Genetic reprogramming (de la Torre and Chin, 2021) could be targeted at enhancing traits such as cooperation, long-term thinking, empathy, and sustainability-focused decision-making while reducing impulsive or short-sighted behaviors (Sjåstad, 2019) that contribute to ecological and social crises. By carefully reengineering these inherited behaviors and eliminating some, we could create a population that is better adapted to the challenges of living in a complex, technological civilization. Genetic enhancement of human intellectual capacities (Tang et al., 1999) could compensate for the decrease in motivation caused by eliminating greed and excessive need for power as brilliant individuals always have other motivations than money and/or power.

A hypothetical species that has reached a very advanced technological stage could have found a way to overcome IBPs entirely. Such species might have developed the means to manipulate or even eliminate the influence of inherited behaviors through sophisticated technologies. Whether through genetic engineering (Tamura and Toda, 2020), brain-computer interfaces (Nicolas-Alonso and Gomez-Gil, 2012), or other advanced methods, these civilizations could have solved the problems posed by IBPs by partially or completely reprogramming their behavior.

In summary, the future may hold solutions to the problem of incompatible inherited behavior patterns through genetic reprogramming and technological interventions. While these solutions are not yet within reach, they provide hope that humanity can evolve in ways that will better align our innate drives with the demands of a sustainable, technologically advanced civilization.

Last but not least, interfering with the genetic foundations that shape human behavior and identity carries some risks, particularly when considering the complexity of human nature and its ethical dimensions. At the ontological level, the essence of what it means to be human could be at stake. Human identity is built on a mixture of personal experiences, genetics, culture, and choice (Lu et al., 2023). By altering core genetic instincts, such as those related to survival, social behavior, or competition, we could interfere with the unique psychological and existential journeys that define individual lives. Ethically, such interference requires careful consideration. Who decides which traits are to be “improved” or reprogrammed, and on what basis? The pressing need for expertise suggests that this question should be addressed by scientists. Scientists need to determine which traits should be prioritized over others, ensuring that any changes do not alter the definition of what it means to be human more than is necessary to create a sustainable technological civilization.
